# Redox status of high-mobility group box 1 performs a dual role in angiogenesis of colorectal carcinoma

**DOI:** 10.1111/jcmm.12577

**Published:** 2015-06-23

**Authors:** Lingyin Zhu, Lin Ren, Yingxuan Chen, Jingyuan Fang, Zhizheng Ge, Xiaobo Li

**Affiliations:** a State Key Laboratory for Oncogenes and Related Genes, Key Laboratory of Gastroenterology & Hepatology, Ministry of Health, Division of Gastroenterology and Hepatology, Ren Ji Hospital, School of Medicine, Shanghai Jiao Tong University, Shanghai Cancer Institute, Shanghai Institute of Digestive DiseaseShanghai, China; b Department of Gastroenterology, Shanghai Jiao Tong University Affiliated Sixth People’s HospitalShanghai, China; c The First People’s Hospital of LianyungangJiangsu Provence, China

**Keywords:** high-mobility group box 1, tumour angiogenesis, all-thiol form, disulfide form, VEGF-A

## Abstract

During inflammation, high-mobility group box 1 in reduced all-thiol form (at-HMGB1) takes charge of chemoattractant activity, whereas only disulfide-HMGB1 (ds-HMGB1) has cytokine activity. Also as pro-angiogenic inducer, the role of HMGB1 in different redox states has never been defined in tumour angiogenesis. To verify which redox states of HMGB1 induces angiogenesis in colorectal carcinoma. To measure the expression of VEGF-A and angiogenic properties of the endothelial cells (ECs), at-HMGB1 or ds-HMGB1 was added to cell medium, further with their special inhibitors (DPH1.1 mAb and 2G7 mAb) and antibodies of corresponding receptors (RAGE Ab and TLR4 Ab). Also, a co-culture system and conditioned medium from tumour cells were applied to mimic tumour microenvironment. HMGB1 triggered VEGF-A secretion mainly through its disulfide form interacting with TLR4, while co-operation of at-HMGB1 and RAGE mediated migratory capacity of ECs. Functional inhibition of HMGB1 and its receptors abrogated HMGB1-induced angiogenic properties of ECs co-cultured with tumour cells. HMGB1 orchestrates the key events of tumour angiogenesis, migration of ECs and their induction to secrete VEGF-A, by adopting distinct redox states.

## Introduction

High-mobility group box 1 (HMGB1), as an architectural chromatin-binding protein, is composed of two positively charged proximal DNA-binding domains (A box and B box), and a negative charged C-terminal tail 1. Three cysteine residues are encoded within the protein, a single one in B box(C106) and two vicinal cysteines in A box(C23 and C45), all of which are sensitive to redox-dependent modifications. Preserving all three cysteines in their reduced form (*i.e*. all-thiol status), all-thiol HMGB1 (at-HMGB1) is the sole status that instigates chemoattractant activity, recruiting leucocytes in inflammation 2. Once a disulfide bond is formed between C23 and C46 but C106 remaining its thiol form, so-called disulfide HMGB1 (ds-HMGB1), rather than other redox states, is capable of activating the leucocytes and triggering the release of pro-inflammatory cytokines or chemokines 3. Interestingly, the terminally oxidated form of HMGB1 includes sulphonation (SO^3−^) of all three cysteines, which is involved in the resolution of inflammation.

Besides its vital role as inflammatory mediator, HMGB1 is a marker of tumour angiogenesis, as established by gene-expression profiles of endothelial cells (ECs) isolated from freshly resected colorectal carcinoma (CRC) 4. HMGB1 can be extracellularly released by activated macrophages and necrotic cells, while many types of cancer cells can actively secrete HMGB1 5–7 or passively release HMGB1 in the process of dying 8,9. Also, ECs were reported to secrete HMGB1 after activation 10 and, in turn, were they activated by exposure to HMGB1, thus creating a positive feedback loop 11. It has been reported 12 how extracellular HMGB1 promotes angiogenesis *via* mediating secretion of angiogenic cytokines and activation of pro-angiogenic cells. However, few or no studies have focused on which redox status of HMGB1 could affect tumour angiogenesis.

The balance between reduced and oxidized states could be shifted in different diseases, including cancer 13,14, where extracellular redox condition is significantly modulated. Intracellular HMGB1 is predominantly in the reduced status, whereas secreted HMGB1 contains both all-thiol and disulfide-bonded forms 2. As time passes, partial oxidation of at-HMGB1 by reactive oxygen species (ROS) may occur, altering the function of HMGB1 from a chemoattractant to a cytokine in response to infection or sterile injury 1. Further exposure to large amounts of ROS leads to the terminal oxidation of HMGB1 3,15. Of note, ROS including superoxide (O^2−^) and hydrogen peroxide (H_2_O_2_) are found in various tumours, which also contribute to angiogenesis 16. Produced in response to hypoxia, ischemia and induction of pro-angiogenic factors such as VEGF, ROS at low levels can stimulate EC proliferation and migration 17.

In this work, we aimed to determine whether different redox status of extracellular HMGB1 performed distinct roles in angiogenesis of human CRC. We demonstrated how HMGB1 stimulation of ECs led to the release of VEGF-A and enhancement of the cells’ angiogenic properties. To elucidate the role of HMGB1 in tumour angiogenesis, we used a co-culture system that had both human ECs and tumour cells, thus avoiding inter-species complications. The results of the study indicate which redox form of extracellular HMGB1 mediates angiogenesis through VEGF-A, and HMGB1 in different redox states may be a novel therapeutic target for tumour angiogenesis.

## Materials and methods

### Cell culture and reagents

Human CRC HCT116 cell line and human umbilical vein endothelial cells (HUVECs) were obtained from China Center for Type Culture Collection (Beijing, China). Both cell lines were cultivated in RPMI 1640 growth medium supplemented with 10% foetal bovine serum (Invitrogen, California, USA) at 37°C in a humidified atmosphere of 5% CO_2_ and 95% air.

For cell co-culture, HUVECs were seeded onto a six-well transwell apparatus with 0.4 μm pore size at a density of 1 × 10^5^ cells/well (Transwell from Millipore, Massachusetts, USA). The apparatus was laid into a six-well culture plate, which had been plated with HCT116 cells (1 × 10^5^ cells/well), and incubated at 37°C for 4 days. HUVECs and the supernatants from the transwell apparatus were collected for further study.

To prepare conditioned medium (CM) 18, HCT116 cells were washed and incubated with a serum-free medium for 2 hrs when subconfluent. The medium was discarded, and the cells were incubated with a serum-free medium again. After 48 hrs, the CM was harvested and centrifuged to remove debris, filtered through a 0.22 μm filter, and stored at −20°C until use.

Where indicated, recombinant HMGB1 (rHMGB1; Sigma-Aldrich: Munich, Germany), at-HMGB1 and ds-HMGB1 (both from HMGBiotech: Milan, Italy) was added to serum-free medium. According to the protocol described previously 19,20, we generated the anti-HMGB1 monoclonal antibody 2G7 could neutralize both chemoattractant and cytokine activity of HMGB1. To inhibit HMGB1-induced chemoattractant activity, we purchased the anti-HMGB1 monoclonal antibody DPH1.1 from HMGBiotech 2. HMGB1 can act through particular receptors, including receptor for advanced glycation end-products (RAGE) and Toll-like receptors (TLR2 and TLR4). Polyclonal rabbit anti-human HMGB1 antibody and antibodies to RAGE, TLR2, TLR4, VEGF-A and β-actin were all from Cell Signaling Technologies (Massachusetts, USA). H_2_O_2_ (30%) was obtained from BDH Chemicals Ltd (Massachusetts, USA).

### Real-time RT-PCR

Total RNA was extracted from cells with Trizol (Invitrogen), and cDNA was synthesized by the use of reverse transcriptase (PrimeScript TM RT reagent Kit Perfect Real Time, TaKaRa, Dalian, China). The relative content of mRNA was amplified by real-time PCR with SYBR green (SYBR® Premix Ex Taq TM II, TaKaRa). PCR was initiated by a 30 sec. denaturation at 95°C, followed by 40 cycles of 95°C for 5 sec. and 60°C for 30 sec. Gene expression was normalized to β-actin mRNA expression and presented as fold-change compared to that of the control experiments.

### Protein extraction and Western blot analysis

Total cellular protein was isolated from fresh tissues or cells with a lysis buffer containing PMSF and RAPI. Protein concentrations in the supernatants were measured with a Bio-Rad protein determination kit (Bio-Rad, California, USA). Protein samples were loaded onto a 10% SDS-PAGE, transferred onto PVDF membranes, and blocked with non-fat milk for 1 hr at room temperature. The membranes were incubated overnight at 4°C with a primary antibody. The membranes were incubated with horseradish peroxidase–conjugated secondary antibodies for 1 hr at room temperature. Immunodetection was performed with Super Signal® West Femto Maximum Sensitivity Substrate (Thermo Fisher, Massachusetts, USA) and exposure to Biomax MR Film (Kodak, Shanghai, China).

### ELISA

VEGF-A concentrations in cell culture supernatants were determined by the use of an ELISA kit (R&D Systems, Minnesota, USA) according to the manufacturers’ instructions, detecting specifically human VEGF121 and VEGF165. The supernatants from co-cultured HUVECs and CM of HCT116 cells were assayed for HMGB1 secretion by ELISA according to the protocol from the producer (Jhbg-Bio, Beijing, China).

### Silencing in HCT116 and HUVEC cell lines

Cells were transiently transfected by the use of Lipofectamine2000 (Invitrogen), with small interfering RNA (siRNA), which was designed and synthesized by GenePharma, Shanghai, China, for knockdown of HMGB-1, RAGE, TLR2 and TLR4. Culture medium was exchanged for serum-free medium after 6 hrs transfection. Cells and culture medium were harvested 24 hrs later. Total cell lysates were prepared for the confirmation of knockdown by the use of RT-PCR.

### *In vitro* invasion and migration assay

Cellular invasion was quantified with a Matrigel chamber assay. Cells (4 × 10^4^ per upper chamber) in serum-free medium were seeded onto Matrigel-coated filters (Millipore). In the lower chambers, growth medium plus 30% foetal bovine serum served as the chemoattractant. After 36 hrs of culture, non-invading cells in the upper chamber were removed with cotton swabs. Invading cells in the lower chamber were fixed with 4% formalin and stained with 0.1% crystal violet. The number of the cells from five microscope fields per filter was counted (original magnification, ×100).

The migration ability of cells was assayed in a monolayer denudation assay. The confluent cells were wounded by scraping with a 100 μl pipette tip. The cultures were washed twice with PBS. Serum-free medium was added, and the cells that migrated into the denuded area were photographed (original magnification, ×100). Wound width was measured at the beginning of the assay and after 24 hrs, and wound closure was expressed relative to cells not exposed to rHMGB1.

### Aortic ring assay

As has been described 21, aortas were harvested from 8-week-old Sprague–Dawley rats and sectioned into several pieces (aortic rings). The rings were placed in 48-well culture plates coated with Matrigel (BD Biosciences, California, USA). rHMGB1, antibodies or CM was added to each well in a final volume of 200 μl culture medium. The plates were incubated at 37°C, and media were changed every 2 days for 1 week. The angiogenic sprouting from aortic rings was examined in five rings per group in each assay. Each aortic ring was photographed (original magnification, ×40), and sprouts areas were quantified with Wimasis Image Analysis (http://www.wimasis.com/en/).

### Tubular formation assay

Matrigel was pipetted into 48-well plates and polymerized for 30 min. at 37°C. HUVEC cells were seeded onto the layer of Matrigel at a density of 2 × 10^4^ cells/well in a final volume of 200 μl culture medium. Matrigel cultures were incubated at 37°C for 12 hrs. After being photographed (original magnification, ×100), HUVEC branching loops and nets were estimated with an optical imaging technique provided by Wimasis Image Analysis (http://www.wimasis.com/en/). The experiment was performed in triplicate and repeated three times.

### Statistical analysis

Statistical analysis was carried out with SPSS 18.0 (SPSS, Inc., Chicago, IL, USA); a *P*-value of <0.05 was considered statistically significant. Results are expressed as the mean ± S.E.M., analysed with *t*-test or Mann–Whitney *U*-test.

## Results

### HMGB1 induced VEGF-A secretion mainly *via* its disulfide-bonded form

**Figure 1:**
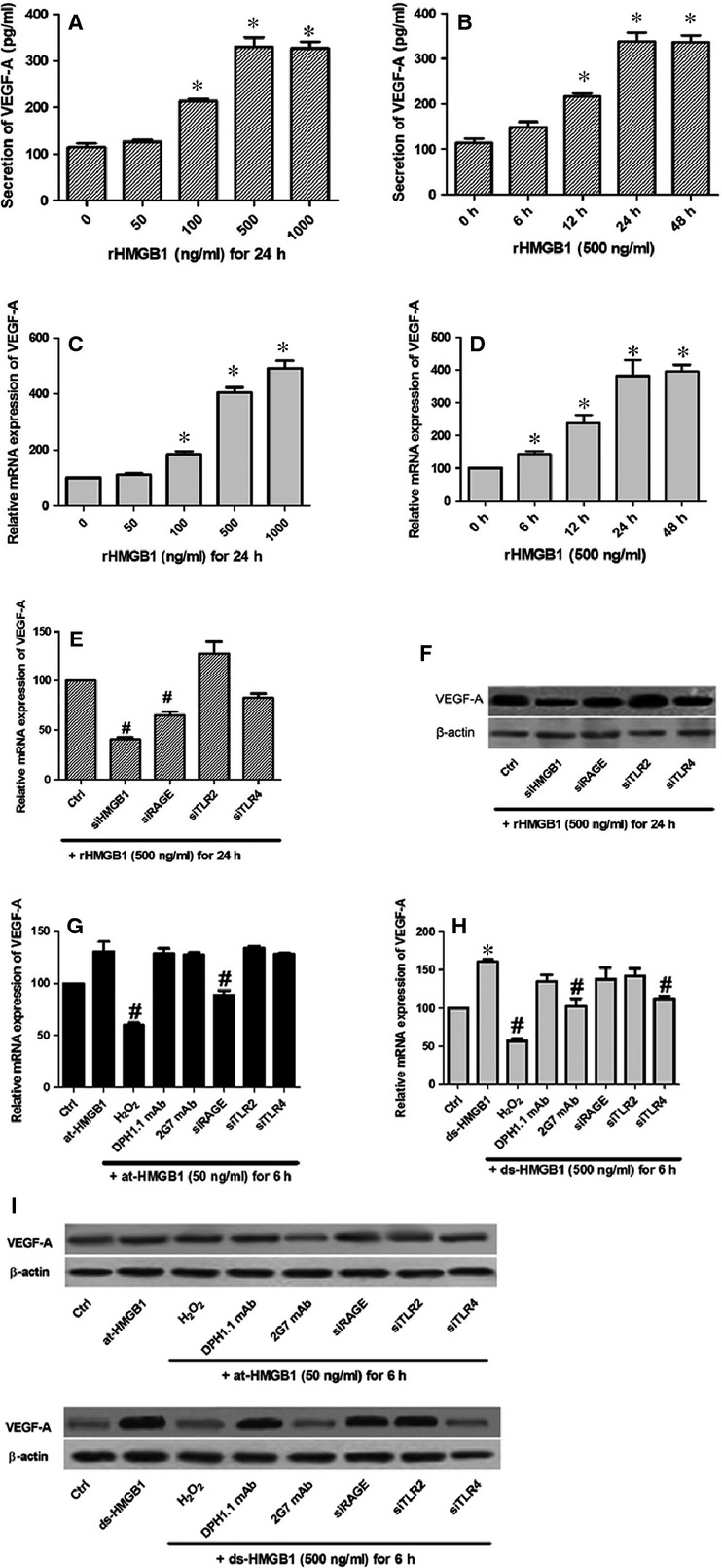
HMGB1, especially its disulfide-bonded form, could regulate the release of VEGF-A. Quantities of VEGF-A expressed after stimulation of HUVECs with increasing concentrations of rHMGB1 (A and C) or duration of stimulation (B and D). (E) At mRNA level, VEGF-A expression was significantly reduced *via* siRNA inhibition of HMGB1 and RAGE. (F) At protein level, siRNA targeting HMGB1, RAGE and TLR4 had an obvious influence on VEGF-A secretion. (G) at-HMGB1 (50 ng/ml) slightly up-regulated VEGF-A expression (*P* > 0.05), which was reversed by effects of H_2_O_2_ and si-RAGE. (H) Oxidation and inhibition by 2G7 mAb (1 μg/ml) or si-TLR4 significantly down-regulated the VEGF-A expression induced by ds-HMGB1 (500 ng/ml). For terminally oxidation, at-HMGB1 or ds-HMGB1 was previously exposed to H_2_O_2_ (50mM) for 1 hr. (I) The similar VEGF-A reduction by H_2_O_2_, 2G7 mAb or si-TLR4 was determined with Western blot, whereas there displayed no effect of at-HMGB1 or its associated inhibition. *Indicates a *P* < 0.05, representing a comparison of cells not exposed to rHMGB1, at-HMGB1 or ds-HMGB1. #Compared with control siRNA-treated cells, *P* < 0.05. All the data were mean ± S.E.M. of three separate experiments.

To assess whether HMGB1 could release VEGF-A from HUVECs, we employed RT-PCR and ELISA methods. VEGF-A secretion correlated with increasing concentrations of rHMGB1 and duration of exposure to rHMGB1 (Fig.1A–D). Medium from untreated HUVEC spheroids contained low levels of VEGF-A (114.4 ± 15.4 pg/ml); amounts increased in a dose-dependent manner by stimulation with 500–1000 ng/ml rHMGB1. After a 12-h stimulation with rHMGB1 (500 ng/ml), VEGF-A secretion increased significantly, to 216.0 ± 12.8 pg/ml, but from 24–48 hrs, it stabilized at 336.7 ± 25.2 to 338.6 ± 33.6 pg/ml.

We applied specific siRNA for HMGB1 and its three receptors (RAGE, TLR2, and TLR4) to HUVECs, resulting in a marked reduction of HMGB1 and receptors expression (60–80%). Expression levels of VEGF-A were reversed through the siRNA targeting HMGB1 and RAGE rather than TLR2 (Fig.1E and F). Interestingly, si-TLR4 significantly decreased VEGF-A protein level, but not mRNA level. Different redox states of HMGB1 were applied to HUVECs for further observation. at-HMGB1 failed to obviously induce expression levels of VEGF-A (Fig.1G and I). However, ds-HMGB1 significantly increased VEGF-A secretion, and effects of H_2_O_2_, 2G7 mAb and si-TLR4 were all capable of inhibiting its secretion (Fig.1H and I).

### Different redox states of HMGB1 modulated each step of the angiogenesis process

**Figure 2:**
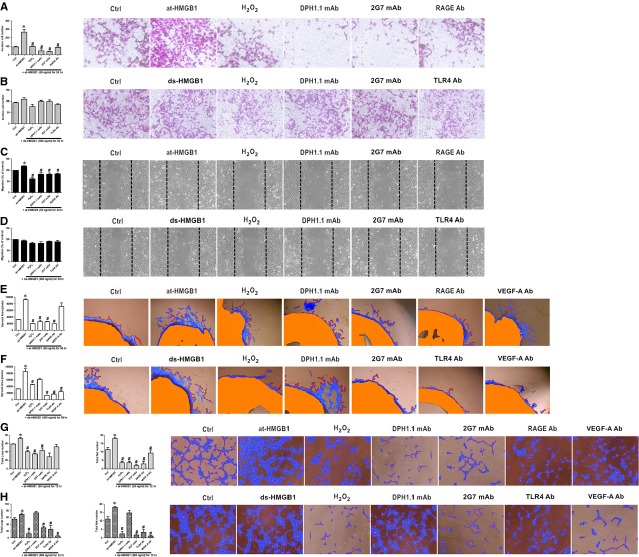
HMGB1 influenced angiogenic properties *via* different redox states. Exposure of HUVECs to 50 ng/ml at-HMGB1 promoted invasion of EC in Matrigel-coated chambers (A) as well as its migration in scratch wound assay (C). ds-HMGB1 (500 ng/ml) as well as its associated inhibition failed to influence EC to invade (B) or migrate (D). (E and F) Aortic rings were embedded in Matrigel containing at-HMGB1 (50 ng/ml) or ds-HMGB1 (500 ng/ml). Sprouts area from the rings was evaluated in the absence or presence of DPH1.1 mAb (0.5 μg/ml), 2G7 mAb (1 μg/ml), antibodies to RAGE, TLR4 and VEGF-A (all in concentration of 10 μg/ml). For terminally oxidation, at-HMGB1 or ds-HMGB1 was previously exposed to H_2_O_2_ (50mM) for 1 hr. (G and H) Similar treatment was applied in a HUVEC tube formation assay. Representative photos of three independent experiments are shown. *Compared with cells not exposed to at-HMGB1 or ds-HMGB1 (Ctrl), *P* < 0.05. #Compared with cells induced by at-HMGB1 or ds-HMGB1 but without antibody-treatment, *P* < 0.05.

Angiogenesis begins with the degradation of the basement membrane by activated ECs, which can migrate and proliferate, leading to the formation of solid sprouts into the stroma. Vascular loops then are formed, and capillary tubes develop 22. at-HMGB1 reacted with HUVECs resulted in the increase in cellular migratory capacity (Fig.2A and C), on the contrary, oxidation and inhibition by DPH1.1 mAb, 2G7 mAb or RAGE Ab neutralized the activity of at-HMGB1. However, ds-HMGB1 and its associated inhibition (including H_2_O_2_, DPH1.1 mAb, 2G7 mAb and TLR4 Ab) had no effect on ECs to invade and migrate (Figure 2B, 2D).

On the one hand, exposure to at-HMGB1 promoted vessels to sprout from aortic rings and tube formation of loops and nets, which could be inhibited by H_2_O_2_, DPH1.1 mAb, 2G7 mAb and RAGE Ab, but not VEGF-A Ab (Fig.2E and G). On the other hand, ds-HMGB1 efficiently stimulated sprouting and tube formation, and this role was abrogated not only *via* H_2_O_2_, 2G7 mAb and TLR4 Ab but also *via* VEGF-A Ab (Fig.2F and H).

### HMGB1 plays a role in tumour angiogenesis

**Figure 3:**
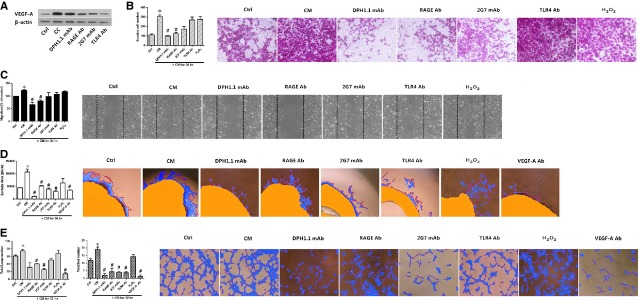
HMGB1 induced tumour angiogenesis. (A) HUVECs were co-cultured with HCT116 cells for 4 days in the absence or presence of the four HMGB1-associated antibodies, involving DPH1.1 mAb (0.5 μg/ml), 2G7 mAb (1 μg/ml), RAGE Ab (10 μg/ml) and TLR4 Ab (10 μg/ml). The supernatants were collected and analysed for VEGF-A secretion. (Ctrl indicates supernatants from HUVECs cultured alone; CC indicates supernatants co-cultured for 4 days without any inhibition). (B–E) Conditioned medium (CM) from HCT116 cells was applied to increase angiogenic properties of HUVECs. However, CM interfered with the four HMGB1-associated antibodies and VEGF-A Ab (10 μg/ml) had a counter effect in distinct conditions. Representative photos of three independent experiments are shown. *Compared with cells not treated by CM (Ctrl); *P* < 0.05. #Compared with CM-induced cells without any inhibition, *P* < 0.05.

To further understand the contribution of extracellular HMGB1 to tumour angiogenesis, we co-cultured ECs and carcinoma cells to mimic a tumour microenvironment. A low amount of HMGB1 was present in supernatants from HUVECs cultured alone (58.0 ± 8.0 ng/ml), which significantly increased to 424.7 ± 26.7 ng/ml after a 4-day stimulation with HCT116 cells. On the basis of reported work, HMGB1 secretion from carcinoma cells 5–7 might have infiltrated into upper chambers, and other pro-inflammatory cytokines from lower chambers could activate ECs to release extracellular HMGB1 10. In experiments to further help determine whether HMGB1 regulates VEGF-A in tumour angiogenesis, we applied the associated antibodies to co-cultured ECs, and VEGF-A concentrations were decreased by 2G7 mAb, TLR4 Ab and RAGE Ab, as demonstrated in Western blot analyses (Fig.3A). Sprouting activity and tube formation were also significantly blocked by VEGF-A Ab (Fig.3D and E).

To determine whether HMGB1 could induce tumour angiogenesis, we incubated HUVECs with CM from HCT116 cells in a tumour-like microenvironment (Fig.3B–E). We starved HCT116 cells, which have relatively high secretion levels of HMGB1, for 24 hrs (261.7 ± 7.1 ng/ml). Conditioned medium from the HCT116 cells produced a significant increase in cellular invasiveness and migration of the HUVECs, while its activity was abrogated by DPH1.1 mAb and RAGE Ab (Fig.3B and C). The sprouts from aortic disks were induced by CM, which was inhibited by antibodies of DPH1.1, 2G7, RAGE and TLR4 (Fig.3D). When HUVEC spheroids were incubated with medium that blocked the expression of at-HMGB1, ds-HMGB1, RAGE and TLR4, the number of loops and nets in tubules was decreased (Fig.3E). Attentionally, oxidation of CM did not inhibit any step of the angiogenesis process.

## Discussion

As a crucial mediator in pathological angiogenesis, VEGF-A is largely responsible for vascularization in malignant tumours 23, and HMGB1 has been implicated in VEGF-A production in several non-malignant circumstances 24–27. However, the relevance between HMGB1 and VEGF-A in tumour angiogenesis has not been well studied. During the development of inflammation, at-HMGB1 does not interfere with ds-HMGB1 in cytokine stimulation; in the meanwhile, ds-HMGB1 does not compete with at-HMGB1 for cell migration. Such two functions of HMGB1 rely on separable receptors. On the one hand, both *in vitro* and *in vivo* 28,29, the chemoattractant activity of HMGB1 appears to involve RAGE because it is inhibited by anti-RAGE antibodies, and the migration of RAGE−/− cells is severely impaired. Also, at-HMGB1 binding the abundant chemokine CXCL12 can act through CXCR4 30, and RAGE is necessary for HMGB1-induced CXCL12 secretion 31. On the other hand, the cytokine-like function of ds-HMGB1 requires the interaction with TLR4, resulting in NF-kB activation 1. Interestingly, different redox status of HMGB1 could have similar influence on tumour angiogenesis according to our results. at-HMGB1 stimulated ECs into migration, and ds-HMGB1 induced the secretion of VEGF-A, both of which contributed to neovascularization.

Our study showed that rHMGB1 triggered the up-regulation of VEGF-A in a dose- and time-dependent manner, which could be down-regulated at protein level *via* inhibiting expression of HMGB1, RAGE and TLR4. Notably, it was ds-HMGB1 rather than at-HMGB1 that promoted VEGF-A secretion, and this effect could be neutralized by antibodies of ds-HMGB1 or TLR4. Antibody targeting at VEGF-A inhibited the vessel sprouting and tubular formation induced by ds-HMGB1, but not those by at-HMGB1. In tumour environment, VEGF-A Ab led to the similar inhibition of angiogenesis, and the increasing secretion of VEGF-A was inhibited by antibodies of ds-HMGB1, TLR4 and RAGE. Together, these data suggest a more dominant role for ds-HMGB1 in mediating the release of VEGF-A through its specific receptor TLR4. RAGE, a multi-ligand receptor, also involved the mediation of VEGF-A, but without evidence that at-HMGB1 could interact with RAGE to induce VEGF-A secretion.

Indeed, RAGE and TLR4 signalling can both converge to the activation of NF-kB 12, leading to the transcription and the activation of pro-angiogenic or pro-inflammatory target genes. Although it is well-defined that HMGB1 can signal through TLR4, data supporting this in ECs are limited. As van Beijnum *et al*. 11 reported, exposure of ECs to rHMGB1 resulted in increased expression both of TLR4 and RAGE, further amplifying the pro-angiogenic response; however, the migration of ECs was blocked by siRNA and antibodies targeting HMGB1 and RAGE, but not TLR4. Furthermore, pre-stimulation of endothelial progenitor cells (EPCs) with rHMGB1 could enhance the recruitment of EPCs for tumour neovascularization, and EPC migration was blocked by RAGE, but not by anti-TLR antibodies 32. In this study, we demonstrated that HMGB1 significantly improved the migratory capacity of ECs through its reduced form, and RAGE is the vital receptor for the pro-angiogenic activity of at-HMGB1.

Consistent with our observation, HMGB1 has been regarded as an inducer for EC proliferation, sprouting and migration 33. Tube formation, an angiogenic phenotype of ECs, was enhanced by HMGB1 treatment, but was impaired by neutralization of endogenous HMGB1 34. In several animal models, blockade of HMGB1 has decreased pathologic angiogenesis and the inhibition of tumour growth and metastasis 11,33,35. Thus, it seems reasonable that HMGB1 has become an attractive target for the suppression of tumour angiogenesis. We applied particular inhibitors of HMGB1 in different redox forms (*i.e*. DPH1.1 mAb and 2G7 mAb) and antibodies of their corresponding receptors (*i.e*. RAGE Ab and TLR4 Ab) so that angiogenic properties of ECs could be efficiently blocked. Attractively, exposure to H_2_O_2_ made HMGB1 inactive, both as inducers for VEGF-A secretion and EC migration; however, H_2_O_2_ failed to prevent angiogenesis of ECs cultured in cancer CM. The balance between the roles of all-thiol and disulfide HMGB1 significantly depends on the extracellular environment 36. Initially secreted in reduced forms, HMGB1 becomes oxidized in the extracellular space, and the redox status of HMGB1 is a reversible process 37. Reactive oxygen species abrogate both pro-inflammatory and chemotactic activities of HMGB1 by terminally oxidizing its cysteines to sulfonates 3. Alternatively, there are antioxidants to inhibit HMGB1 release, leading to the suppression of cell death and inflammatory responses 38; furthermore, ROS has been considered as a promoter for tumour angiogenesis *in vivo*, which could be attenuated by different antioxidants 16. Taken together, we presume that antioxidants or inhibitors against different redox forms of HMGB1 should be better methods than oxidants to inhibit the HMGB1-induced tumour angiogenesis.

In conclusion, our available experimental evidence suggest a dual role of HMGB1 in tumour angiogenesis: recruitment of ECs and their activation to secrete pro-angiogenic factor VEGF-A, which depends on distinct redox states of the same protein. HMGB1, combined with its receptors RAGE and TLR4, appears to be an attractive target for treatment of tumour angiogenesis *via* neutralization by specific antibodies. However, these proposed therapeutic benefits will require extensive research to establish their validity.
